# Exploring Genetic Associations Between Autoimmune Diseases and Pathological Scars: A Bidirectional Mendelian Randomization Analysis

**DOI:** 10.1111/jocd.71128

**Published:** 2026-08-19

**Authors:** Xueqi Wang, Xianglin Dong

**Affiliations:** ^1^ Department of Plastic Surgery The First Affiliated Hospital of Xinjiang Medical University Urumqi China

**Keywords:** autoimmune diseases, causal inference, mendelian randomization, pathological scars

## Abstract

**Background:**

Autoimmune diseases and pathological scars share inflammatory and fibroproliferative features, but observational evidence cannot establish whether their co‐occurrence reflects causation or shared biology. We used bidirectional Mendelian randomization (MR) to test genetic associations between clinically defined autoimmune diseases and pathological scars.

**Methods:**

We analyzed 10 autoimmune diseases and 2 pathological scar phenotypes (keloids and hypertrophic scars) in both directions, comprising 40 primary IVW tests. Autoimmune‐disease instruments were selected at *p* < 5 × 10^−8^ and scar instruments at *p* < 5 × 10^−6^ because few genome‐wide significant scar loci were available. Independent SNPs were obtained by LD clumping (*r* ^2^ < 0.001; 10 000 kb), and variants with *F* ≤ 10 were excluded. Multiplicative random‐effects inverse‐variance weighted (IVW) analysis was primary, with MR‐Egger, weighted median, mode‐based estimators, Cochran's *Q*, MR‐Egger intercept, leave‐one‐out analysis, MR‐PRESSO, and radial MR as sensitivity analyses. Benjamini–Hochberg false discovery rate (FDR) correction was applied across the 40 primary tests.

**Results:**

Three associations met nominal significance based on raw *p* values: hypertrophic scars with celiac disease (OR = 1.099, 95% CI 1.036–1.165, *p* = 0.00157; 2 SNPs), rheumatoid arthritis with hypertrophic scars (OR = 1.119, 95% CI 1.026–1.220, *p* = 0.0107; 19 SNPs), and type 1 diabetes with keloids (OR = 1.212, 95% CI 1.048–1.401, *p* = 0.0090; 12 SNPs). None survived FDR correction (minimum FDR‐adjusted *p* = 0.0628). The remaining 37 primary tests had raw *p* ≥ 0.05.

**Conclusion:**

The analyses identified only nominal associations that were not robust to multiple‐testing correction and therefore do not provide reliable evidence of causality. The results offer an initial framework for investigating potential shared genetic signals and should be treated as hypothesis‐generating pending independent validation.

**Abbreviations**
ADs: autoimmune diseases
CD: Crohn's disease
CI: confidence interval
GWAS: Genome‐Wide Association Study
IVs: instrumental variables
IVW: inverse variance weighted
LD: linkage disequilibrium
LOO: leave‐one‐out (analysis)
MAF: minor allele frequency
MR: mendelian randomization
MS: multiple sclerosis
OR: odds ratio
PBC: primary biliary cholangitis
RA: rheumatoid arthritis
SLE: systemic lupus erythematosus
SNP: single nucleotide polymorphism
T1D: type 1 diabetes
UC: ulcerative colitis

## 1 Introduction

A diminished tolerance to self‐antigens is the primary characteristic of autoimmune diseases (ADs), a complex set of disorders. These conditions arise when the body generates numerous autoantibodies and immune complexes, which ultimately results in dysfunction across multiple organs and systems [1]. These diseases not only severely impair patients' quality of life and reduce their ability to participate in society but also impose a significant economic burden on global healthcare systems [2]. Abnormalities in the wound healing process can trigger fibroproliferative skin conditions, specifically referred to as keloids and hypertrophic scars [3]. These lesions are pathologically defined by an overabundance of collagen buildup. Clinically, they not only affect patient aesthetics but are also frequently accompanied by physical discomforts, such as intense pruritus and pain [4]. Keloids can extend beyond the original injury boundaries, whereas hypertrophic scars, though often red and raised, remain confined to the initial injury site [5]. These scars not only affect appearance but can also lead to functional impairment and psychological distress.

Immune dysregulation and fibrosis provide a biological rationale for studying autoimmune diseases together with pathological scars [6], but shared pathways alone do not demonstrate causality. In keloids, interactions among immune cells, fibroblasts, and profibrotic signaling pathways, including TGF‐β1/Smad and PI3K/AKT/mTOR, have been described [7, 8]. Recent MR studies have also examined inflammatory cytokines and immune‐cell phenotypes in relation to hypertrophic scars. These studies focus on molecular intermediates rather than clinically defined autoimmune diseases, leaving uncertainty about whether inherited liability to autoimmune disease is associated with scar risk or vice versa.

Although these observations point to a shared inflammatory and fibroproliferative basis, the available evidence is almost entirely observational. Cross‐sectional and case‐series data cannot separate genuine causal effects from confounding, and several plausible confounders are difficult to eliminate: autoimmune diseases and pathological scars share demographic risk factors such as female predominance, and patients with autoimmune disease are commonly treated with glucocorticoids and immunosuppressants that themselves modify fibrosis. Reported associations have also been inconsistent in direction and magnitude across studies, and it remains unclear whether autoimmune disease promotes pathological scarring, whether scar‐prone biology influences autoimmune risk, or whether the relationship is bidirectional. Consequently, it is still uncertain whether the co‐occurrence of these conditions reflects causation or merely correlation.

MR uses germline variants as instrumental variables and can reduce, although not eliminate, confounding and reverse causation when its assumptions are satisfied [9, 10]. The primary objective of this study was to test whether genetic liability to 10 clinically defined autoimmune diseases is associated with the risk of keloids or hypertrophic scars. The secondary objective was to test the reverse direction, from genetic liability to each scar phenotype to autoimmune‐disease risk. We predefined directional hypotheses that autoimmune liability may increase pathological‐scar risk and that scar liability may be associated with autoimmune‐disease risk because both directions and 20 phenotype pairs were tested, all findings were evaluated under multiple‐testing correction and interpreted cautiously.

## 2 Materials and Methods

### 2.1 Data Sources

Keloid summary statistics were obtained from the GWAS Catalog (GCST90476200), hypertrophic‐scar data from FinnGen release 12 (L12_HYPETROPHICSCAR), and autoimmune‐disease data from IEU OpenGWAS or GWAS Catalog studies listed in Table 1 [11, 12]. The 10 autoimmune diseases were Crohn's disease, ulcerative colitis, primary biliary cholangitis, systemic lupus erythematosus, celiac disease, psoriasis, ankylosing spondylitis, multiple sclerosis, type 1 diabetes, and rheumatoid arthritis. All datasets were derived predominantly from participants of European ancestry. Because only summary‐level data were available, exact participant overlap among FinnGen, IEU OpenGWAS, and GWAS Catalog studies could not be determined. The use of distinct source studies makes complete overlap unlikely, but partial overlap remains possible and could bias estimates toward observational associations, particularly for weaker instruments. F statistics were used to assess instrument strength; they do not remove sample‐overlap bias. Dataset details are provided in Table 1.

**TABLE 1:** Basic information and data sources of the study.

Phenotype	GWAS ID	PMID	Population	Cases (*n*)	Controls (*n*)
Systemic lupus erythematosus	GCST003156	26 502 338	European	5201	9066
Psoriasis	GCST90038681	33 959 723	European	5427	479 171
Type 1 diabetes	GCST90018925	34 594 039	European	6447	451 248
Primary biliary cholangitis	GCST90061440	34 033 851	European	8021	16 489
Rheumatoid arthritis	GCST90018910	34 594 039	European	8255	409 001
Ulcerative colitis	GCST90018933	34 594 039	European	5371	412 561
Celiac disease	ieu‐a‐276	20 190 752	European	4533	10 750
Crohn's disease	ieu‐a‐30	26 192 919	European	5956	14 927
Multiple sclerosis	GCST003566	27 386 562	European	4888	10 395
Ankylosing spondylitis	GCST90476232	39 024 449	European	1637	448 993
Keloid	GCST90476200	39 024 449	European	2079	444 929
Hypertrophic scar	L12_HYPETROPHICSCAR	NA	European	2068	465 673

### 2.2 Study Design

**FIGURE 1:**
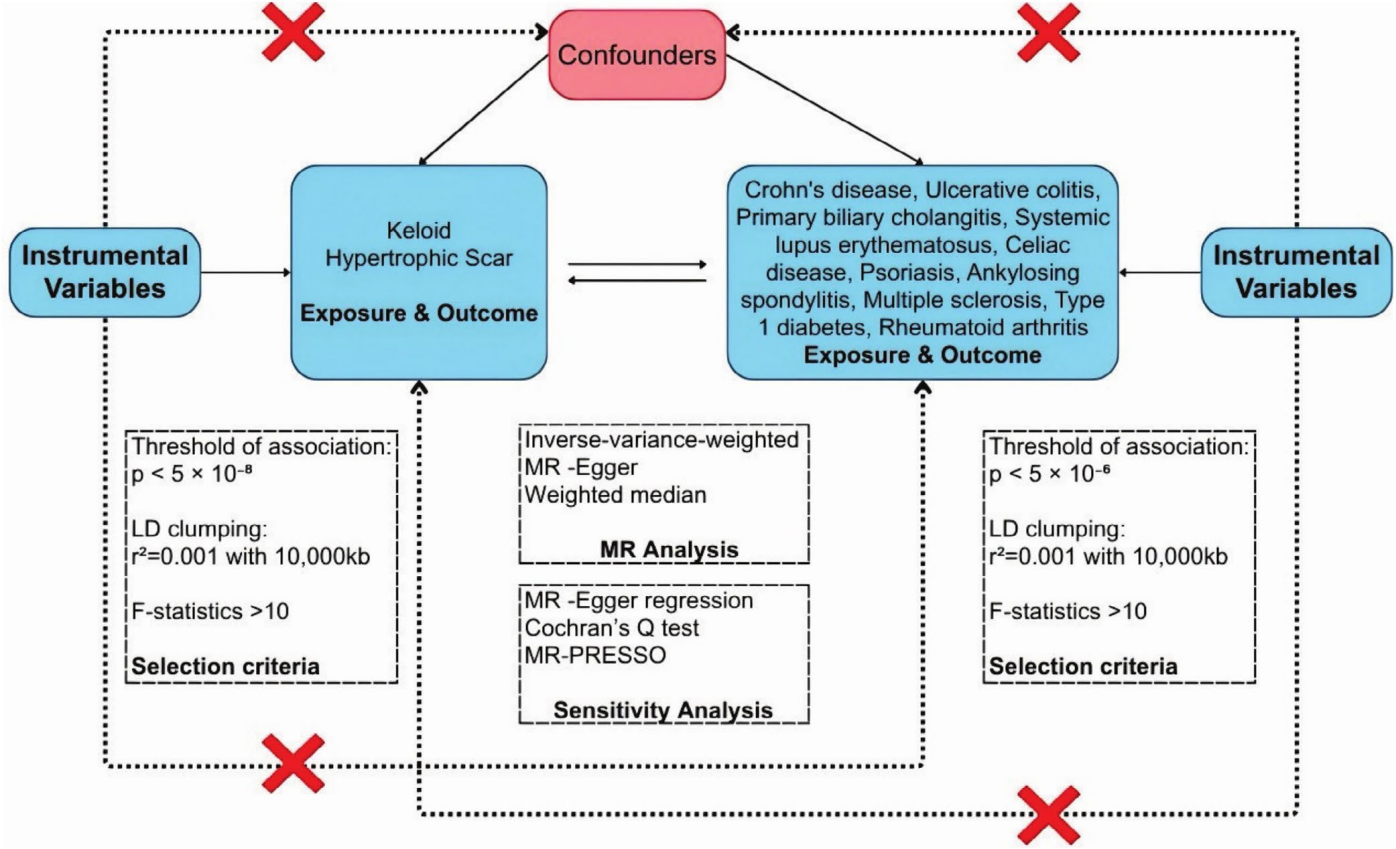
Bidirectional two‐sample Mendelian randomization study design. Genetic instruments were clumped at *r* ^2^ < 0.001 within 10 000 kb; autoimmune‐disease instruments used *p* < 5 × 10^−8^ and scar instruments used *p* < 5 × 10^−6^.

This bidirectional two‐sample MR study used publicly available summary‐level GWAS data and was reported with reference to STROBE‐MR recommendations. Ethical approval and informed consent were obtained by the original studies; no additional participant‐level approval was required. Three core assumptions were evaluated in practice. Relevance was addressed through exposure‐association thresholds, LD clumping, and exclusion of SNPs with *F* ≤ 10. Independence was supported by restricting analyses to predominantly European ancestry, LD clumping, and screening candidate variants in the GWAS Catalog for reported associations with plausible confounders. The exclusion‐restriction assumption was assessed by excluding variants strongly associated with the outcome and by applying MR‐Egger, MR‐PRESSO, heterogeneity tests, radial MR, and leave‐one‐out analyses. These procedures reduce but cannot eliminate violations, especially unmeasured or balanced pleiotropy [13, 14]. The study flowchart is shown in Figure 1.

### 2.3 Selection of Instrumental Variables

For autoimmune‐disease exposures, SNPs were selected at *p* < 5 × 10^−8^. For pathological‐scar exposures, *p* < 5 × 10^−6^ was used because the available scar GWAS contained too few genome‐wide significant loci for the planned bidirectional analyses. This increased instrument availability but also increased susceptibility to weak‐instrument bias, horizontal pleiotropy, and winner's curse; scar‐based results were therefore treated as exploratory. Independent variants were obtained by LD clumping (*r* ^2^ < 0.001; 10 000‐kb window) using the 1000 Genomes European reference panel [15]. F statistics were calculated as beta^2^/SE^2^, and SNPs with *F* ≤ 10 were excluded [16, 17]. Candidate variants were queried in the GWAS Catalog, and SNPs with reported genome‐wide associations with the outcome or plausible confounders were excluded where identified. When an exposure SNP was absent from the outcome dataset, no proxy SNP was introduced; the missing variant was removed. During harmonization, allele effects were aligned to the same effect allele, and ambiguous palindromic SNPs were excluded rather than inferred from allele frequency because effect‐allele frequency information was not consistently available.

### 2.4 MR Analysis and Data Visualization

Analyses were conducted in R 4.3.0 using TwoSampleMR and MR‐PRESSO. The primary estimator was multiplicative random‐effects IVW because it combines SNP‐specific ratio estimates efficiently while allowing residual between‐SNP heterogeneity. A fixed‐effects IVW model was not selected as primary because it assumes homogeneity and can yield overly precise estimates when instruments differ. Weighted‐median estimates were considered more reliable when at least 50% of the total weight was expected to arise from valid instruments; MR‐Egger was used mainly as a directional‐pleiotropy diagnostic because it is less precise and depends on the InSIDE assumption. Simple‐ and weighted‐mode estimates provided additional robustness checks. Effect estimates are reported as odds ratios (ORs) with 95% confidence intervals (CIs). For the 20 phenotype pairs assessed in two directions, 40 primary IVW hypotheses were corrected together using the Benjamini–Hochberg procedure. FDR‐adjusted *p* < 0.05 defined statistical significance; raw *p* < 0.05 with FDR‐adjusted *p* ≥ 0.05 was considered nominal and hypothesis‐generating.

### 2.5 Sensitivity Analysis

Cochran's Q test assessed heterogeneity among SNP‐specific estimates. Directional horizontal pleiotropy was evaluated with the MR‐Egger intercept, and MR‐PRESSO and radial MR were used to identify outlying instruments where the number of SNPs permitted. Leave‐one‐out analyses assessed whether a single variant drove an estimate, and funnel and scatter plots were inspected for asymmetry or influential observations. MR‐Egger has low statistical power, particularly with few instruments, and its validity depends on the InSIDE assumption; MR‐PRESSO is designed to detect outliers and cannot identify every form of balanced or correlated pleiotropy. Consequently, nonsignificant diagnostic tests were not treated as proof that pleiotropy was absent.

## 3 Results

### 3.1 Selection of Genetic Instrumental Variables

For scar‐to‐autoimmune analyses, 19 unique hypertrophic‐scar instruments and 21 unique keloid instruments were available across outcome datasets after clumping, harmonization, GWAS Catalog screening, removal of missing or ambiguous palindromic variants, outcome‐association filtering, and *F* > 10 filtering. The median F statistic was 22.49 (range, 20.84–135.34) for hypertrophic‐scar instruments and 23.22 (range, 21.10–108.52) for keloid instruments. The number retained for each outcome varied because outcome datasets did not contain every exposure SNP. Full SNP‐level characteristics are provided in Tables S1 and S2.

For autoimmune‐to‐scar analyses, exposure‐specific instrument sets ranged from 6 unique SNPs for ankylosing spondylitis to 52 for Crohn's disease. Median *F* statistics ranged from 30.73 for type 1 diabetes to 326.21 for ankylosing spondylitis; the complete exposure‐specific medians and ranges are reported in Tables S3 and S4. The number of SNPs included in an individual MR test varied after outcome matching and harmonization. All retained instruments had *F* > 10.

### 3.2 Genetic Associations of Pathological Scars With Autoimmune Diseases

Across the 20 scar‐to‐autoimmune primary IVW tests, only the hypertrophic scar–celiac disease estimate had a raw *p* < 0.05. This was a nominal two‐SNP result and did not meet the FDR‐adjusted significance criterion (Table 2).

**TABLE 2:** Summary of the 40 primary IVW tests after Benjamini–Hochberg correction.

Exposure	Outcome	SNPs	Raw *p*	FDR *p*
Hypertrophic scar	Systemic lupus erythematosus	11	0.522	0.9027
	Psoriasis	14	0.95	0.959
	Type 1 diabetes	18	0.45	0.9027
	Primary biliary cholangitis	4	0.0865	0.51
	Rheumatoid arthritis	15	0.8283	0.9027
	Ulcerative colitis	15	0.511	0.9027
	Celiac disease	2	0.00157	0.0628
	Crohn's disease	18	0.433	0.9027
	Multiple sclerosis	12	0.808	0.9027
	Ankylosing spondylitis	18	0.796	0.9027
Keloid	Systemic lupus erythematosus	13	0.645	0.9027
	Psoriasis	21	0.697	0.9027
	Type 1 diabetes	20	0.362	0.9027
	Primary biliary cholangitis	6	0.0837	0.51
	Rheumatoid arthritis	20	0.102	0.51
	Ulcerative colitis	20	0.283	0.8708
	Celiac disease	4	0.209	0.7347
	Crohn's disease	19	0.959	0.959
	Multiple sclerosis	16	0.391	0.9027
	Ankylosing spondylitis	21	0.728	0.9027
Systemic lupus erythematosus	Hypertrophic scar	40	0.177	0.7347
Psoriasis		14	0.536	0.9027
Type 1 diabetes		16	0.2	0.7347
Primary biliary cholangitis		36	0.692	0.9027
Rheumatoid arthritis		19	0.0107	0.1427
Ulcerative colitis		16	0.657	0.9027
Celiac disease		12	0.2204	0.7347
Crohn's disease		44	0.0873	0.51
Multiple sclerosis		19	0.474	0.9027
Ankylosing spondylitis		2	0.641	0.9027
Systemic lupus erythematosus	Keloid	42	0.7441	0.9027
Psoriasis		15	0.616	0.9027
Type 1 diabetes		12	0.009	0.1427
Primary biliary cholangitis		40	0.835	0.9027
Rheumatoid arthritis		20	0.593	0.9027
Ulcerative colitis		16	0.413	0.9027
Celiac disease		12	0.346	0.9027
Crohn's disease		44	0.093	0.51
Multiple sclerosis		19	0.801	0.9027
Ankylosing spondylitis		4	0.9517	0.959

### 3.3 Genetic Associations of Hypertrophic Scars With Autoimmune Diseases

**FIGURE 2:**
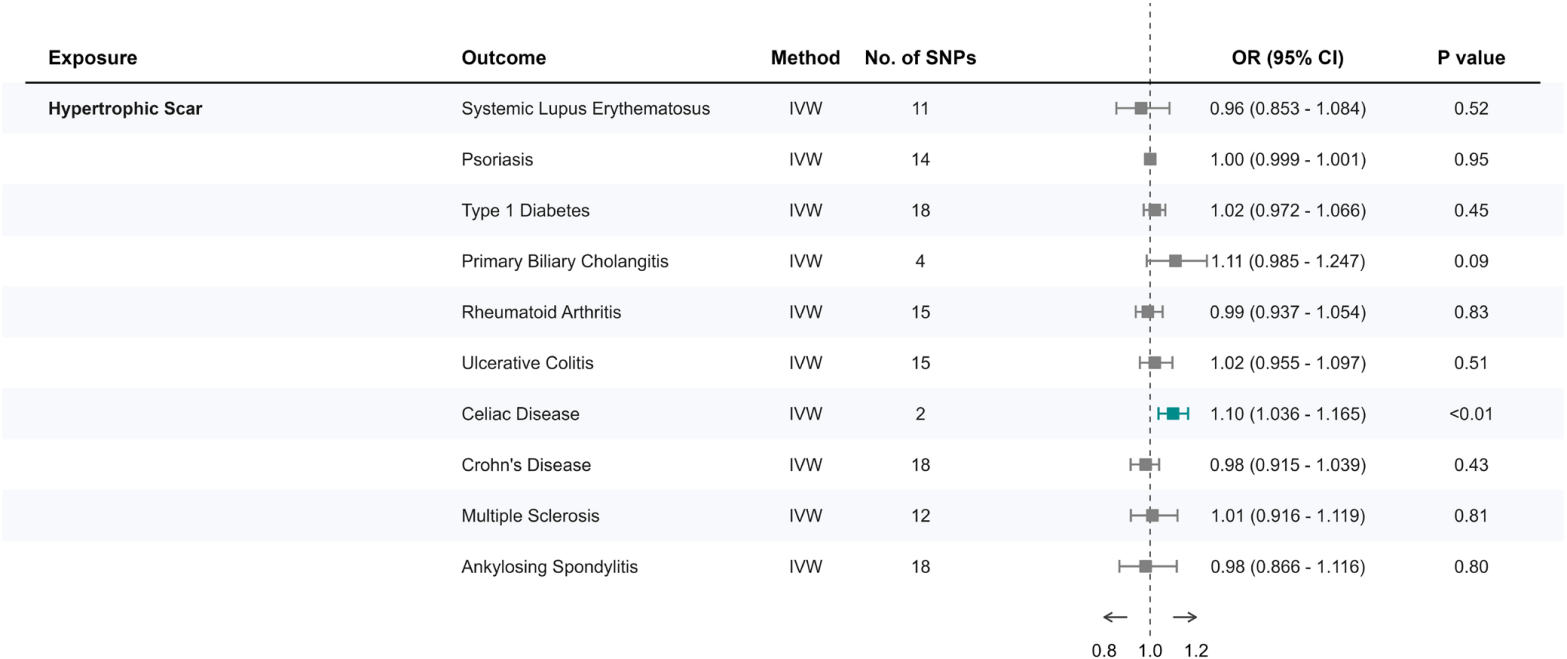
Forest plot of IVW estimates for hypertrophic‐scar liability and 10 autoimmune diseases, using 2–18 SNPs per comparison after harmonization.

Hypertrophic scars showed a nominal association with celiac disease (IVW OR = 1.10, 95% CI 1.036–1.165, *p* < 0.01) (Figure 2). However, this association was based on only two SNPs, which limited the reliability of sensitivity analyses. Importantly, the association did not survive FDR correction and should therefore be interpreted as exploratory. For hypertrophic scars and rheumatoid arthritis, the IVW estimate was not significant (OR = 0.99, 95% CI 0.937–1.054, *p* = 0.83) (Figure 2). Cochran's *Q* test indicated heterogeneity (Table S5), and the MR‐Egger intercept suggested potential horizontal pleiotropy (*p* = 0.04; Table S5). These results indicate that this association should not be interpreted as robust causal evidence.

For the remaining autoimmune outcomes, IVW raw *p* values were ≥ 0.05 (Figure 2 and Table 2). Sensitivity statistics and diagnostic plots are provided in Table S5 and Figures S1–S10. Nonsignificant diagnostic tests do not prove the absence of heterogeneity or pleiotropy.

**FIGURE 3:**
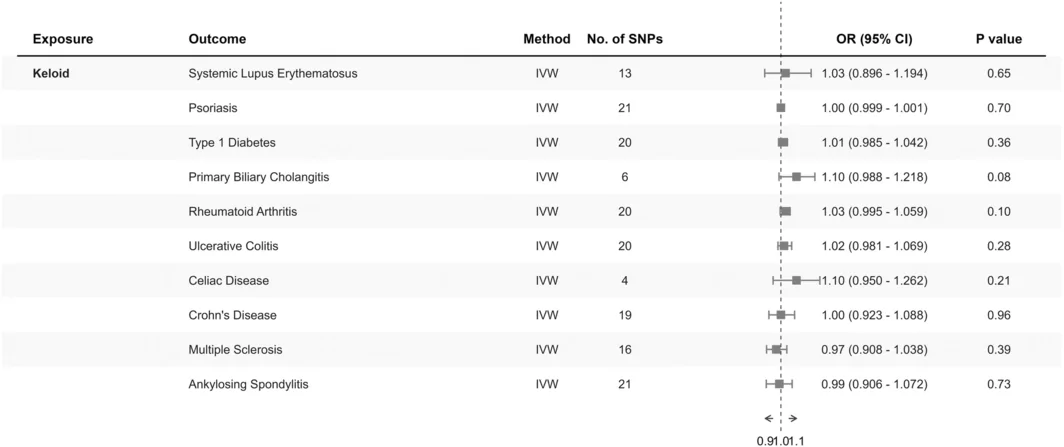
Forest plot of multiplicative random‐effects IVW estimates for keloid liability and 10 autoimmune diseases, using 4–21 SNPs per comparison after harmonization.

Keloids showed no nominal association with psoriasis (IVW OR = 1.00, 95% CI 0.999–1.001, *p* = 0.07) or Crohn's disease (IVW OR = 1.00, 95% CI 0.923–1.088, *p* = 0.96) (Figure 3). Cochran's *Q* test suggested heterogeneity for some analyses (Table S6), but the MR‐Egger intercept test did not indicate clear evidence of horizontal pleiotropy. Overall, these findings do not support a robust causal interpretation.

For the other autoimmune outcomes, IVW raw *p* values were ≥ 0.05 (Figure 3 and Table 2). Complete sensitivity results and diagnostic plots are reported in Table S6 and Figures S11–S20; these analyses did not change the overall null interpretation but cannot exclude all pleiotropy.

### 3.4 Genetic Associations of Autoimmune Diseases With Pathological Scars

Across the 20 autoimmune‐to‐scar primary IVW tests, rheumatoid arthritis–hypertrophic scar and type 1 diabetes–keloid had raw *p* < 0.05, but neither remained significant after FDR correction (Table 2).

### 3.5 Genetic Associations of Autoimmune Diseases With Hypertrophic Scars

**FIGURE 4:**
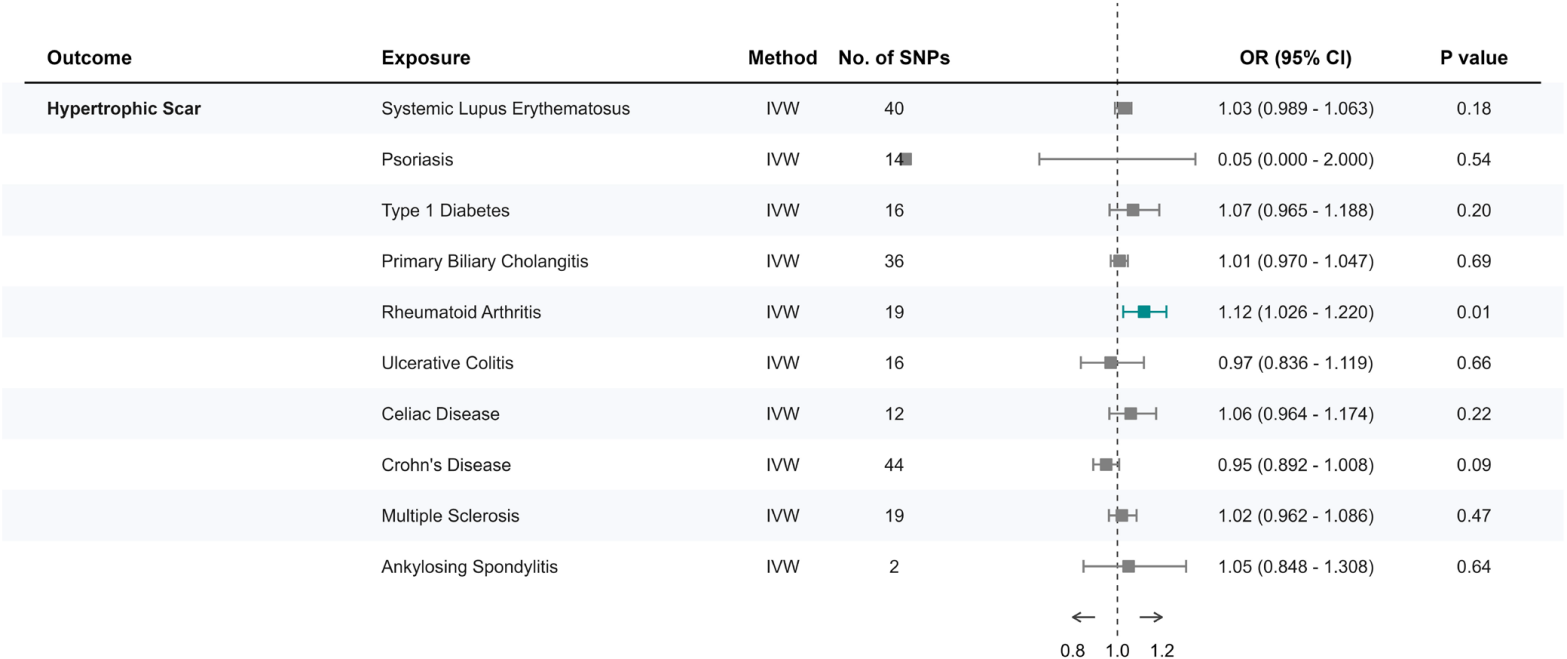
Forest plot of multiplicative random‐effects IVW estimates for 10 autoimmune‐disease liabilities and hypertrophic scars, using 2–44 SNPs per comparison after harmonization.

Rheumatoid arthritis showed a nominal association with hypertrophic scars (IVW OR = 1.12, 95% CI 1.026–1.220, *p* = 0.01) (Figure 4). However, this association did not remain significant after FDR correction. Cochran's *Q* test did not indicate significant heterogeneity, and the MR‐Egger intercept test did not indicate clear evidence of horizontal pleiotropy (Table S7). Leave‐one‐out analysis suggested that the association was not driven by any single SNP, but the finding remains exploratory (Figure S25).

For the other autoimmune‐disease exposures, IVW raw *p* values were ≥ 0.05 (Figure 4 and Table 2). Complete sensitivity results are provided in Table S7 and Figures S21–S30. These null estimates may also reflect limited power and should not be interpreted as evidence that modest effects are absent.

### 3.6 Genetic Associations of Autoimmune Diseases With Keloids

**FIGURE 5:**
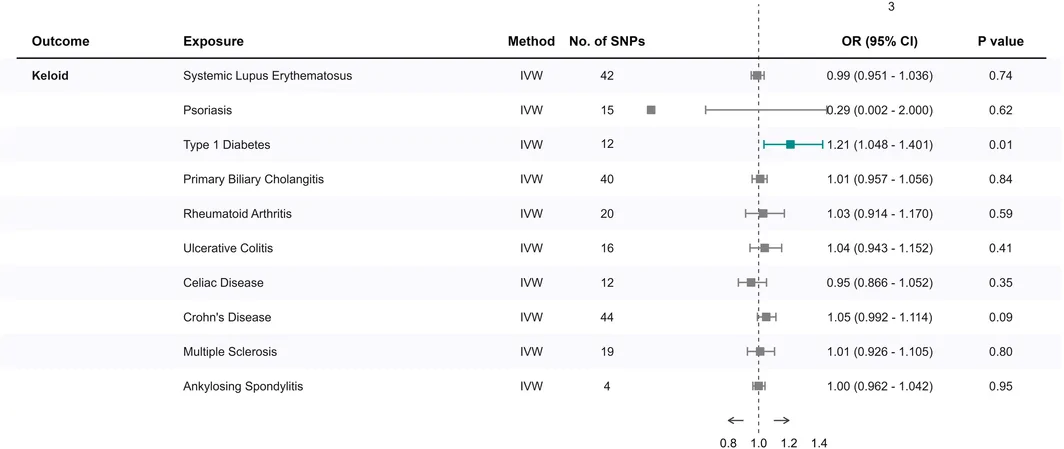
Forest plot of multiplicative random‐effects IVW estimates for 10 autoimmune‐disease liabilities and keloids, using 4–44 SNPs per comparison after harmonization.

Type 1 diabetes showed a nominal association with keloids (IVW OR = 1.21, 95% CI 1.048–1.401, *p* = 0.01) (Figure 5). However, this association did not survive FDR correction. The MR‐Egger intercept did not indicate horizontal pleiotropy, and MR‐PRESSO did not detect outliers (Table S8). Radial MR was used as an additional outlier check and did not materially change the overall interpretation. Leave‐one‐out analysis suggested that the result was not driven by a single SNP (Figure S33).

For the other autoimmune‐disease exposures, IVW raw *p* values were ≥ 0.05 (Figure 5 and Table 2). Complete sensitivity results are provided in Table S8 and Figures S31–S40. Given the small keloid case sample, these null estimates do not exclude modest associations.

### 3.7 FDR Correction

Among the 40 primary IVW tests, three had raw *p* < 0.05. Benjamini–Hochberg correction across all 40 tests yielded no FDR‐adjusted *p* < 0.05; the smallest adjusted value was 0.0628 for hypertrophic scars with celiac disease. Thus, all three signals are nominal and hypothesis‐generating, and the data do not establish a causal association (Table 2).

## 4 Discussion

Although recent MR studies have examined links between specific inflammatory mediators or immune‐cell phenotypes and scarring, to our knowledge this is among the first studies to evaluate genetic causal relationships between a broad panel of clinically defined autoimmune diseases and pathological scars in both directions. Our analysis yielded nominally significant associations of genetically predicted hypertrophic scars with celiac disease, with rheumatoid arthritis with hypertrophic scars, and with type 1 diabetes with keloids. None of these associations, however, remained significant after false discovery rate correction, and they should therefore be regarded as suggestive and hypothesis‐generating rather than as established causal effects. The mechanistic interpretations discussed below are accordingly offered as candidate explanations to be tested in future work, not as confirmed pathways.

Previous MR studies have reported associations of selected inflammatory mediators or immune‐cell phenotypes with scar outcomes [18, 19, 20]. Those findings suggest testable immune–fibrotic pathways but do not establish that clinically defined autoimmune diseases cause pathological scars. Observational similarities, including sex distributions and the fibroinflammatory features of diabetic mastopathy [21, 22, 23], may reflect shared determinants, disease consequences, treatment, or confounding. In the present study, the absence of FDR‐significant results means that these external observations should be viewed as biological context for future hypotheses rather than confirmation of the nominal MR signals.

The formation of pathological scars and autoimmune diseases is both closely associated with inflammatory responses, and their pathological mechanisms share certain similarities. During pathological scar formation, inflammatory cells (such as macrophages and T cells) and inflammatory factors (such as IL‐6 and TNF‐α) play crucial roles in tissue repair [24]. These inflammatory factors not only promote fibroblast proliferation and collagen deposition but may also lead to tissue fibrosis. Similarly, in autoimmune diseases, excessive activation of the immune system and abnormal release of inflammatory factors also induce tissue damage and fibrosis [25]. Compared to normal skin, the histological features of pathological scars show significant immune cell infiltration, such as significantly increased numbers of CD1a (+) Langerhans cells, CD3(+) T lymphocytes, CD68(+) macrophages, and CD20(+) B lymphocytes [26]. This phenomenon of immune cell infiltration is highly consistent with observations in autoimmune diseases, such as the abnormal response of T cells to normal host components [27]. Furthermore, exosomes, mTOR pathway, and Sirtuins proteins exhibit common regulatory mechanisms in keloids and autoimmune skin diseases [28, 29, 30].

Wound healing is a dynamic and overlapping process involving hemostasis, inflammation, proliferation, re‐epithelialization, and remodeling [31]. Throughout these stages, intricate interactions occur between immune cells and fibroblasts, with immune cell infiltration representing a hallmark feature of keloid pathogenesis. Since the 1970s, immunological mechanisms have been proposed in keloid formation [32], and early reports frequently suggested a potential role for autoimmunity. To date, several susceptibility genotypes associated with immune pathways have been identified, including polymorphisms in the genes encoding interleukin‐6 (IL‐6) and transforming growth factor‐beta (TGF‐β) receptors [33]. The preferential recruitment of immune cells is orchestrated through their crosstalk with keloid fibroblasts, thereby modulating the cutaneous repair process [34]. In keloid tissues, increased numbers of mast cells are observed both within and surrounding the lesions, and degranulated mast cells are often found in close contact with activated fibroblasts, indicating direct intercellular communication [35]. As pro‐fibrotic mediators, mast cells infiltrate the wound site during the inflammatory and proliferative phases and play pivotal roles in wound healing by initiating inflammatory responses, promoting re‐epithelialization, and inducing angiogenesis [36]. Immune‐cell infiltration is a hallmark of keloid pathogenesis, and mast cells in particular act as pro‐fibrotic mediators during the inflammatory and proliferative phases of wound healing, where they stimulate fibroblast proliferation and collagen synthesis through TGF‐β1/Smad and PI3K/AKT/mTOR signaling [7, 8]. These shared pathways provide a plausible mechanistic backdrop for the disease‐specific signals discussed below.

The following disease‐specific accounts are offered as hypotheses—intended to explain why particular autoimmune diseases, rather than autoimmunity in general, might map onto particular scar phenotypes—and require direct experimental and clinical testing. Rheumatoid arthritis (RA) may preferentially associate with hypertrophic scars because RA pathobiology includes pathogenic fibroblast‐like synoviocytes, persistent IL‐6/TGF‐β‐shifted inflammation, and the acquisition of mesenchymal/fibrotic features, which is highly compatible with the inflammatory fibroproliferative nature of hypertrophic scars [37]. Type 1 diabetes (T1D) may preferentially associate with keloids because its autoimmune background is accompanied by chronic hyperglycemia and AGEs‐RAGE/TGF‐β‐mediated extracellular matrix remodeling, creating a more direct route to excessive collagen deposition [38]; notably, long‐standing T1D can manifest diabetic mastopathy, a fibroinflammatory lesion with keloid‐like fibrosis. By contrast, the null results for systemic lupus erythematosus (SLE) and psoriasis may reflect both distinct biology and study design limitations: SLE is highly heterogeneous, and fibrosis‐related skin damage is not a uniform whole‐disease phenotype [39]; whereas psoriasis is primarily driven by an IL‐23/IL‐17–keratinocyte axis that overlaps less directly with dermal scar remodeling [40]. These explanations remain biologically plausible rather than definitive, and the absence of significant MR signals for other autoimmune diseases should be interpreted cautiously given phenotype heterogeneity and limited statistical power.

Autoimmune diseases and scar treatment share similarities in the application of glucocorticoids, combination therapies, and immunomodulation. These approaches alleviate symptoms or inhibit fibrosis by anti‐inflammatory and immunosuppressive effects, while also focusing on reducing recurrence risk and adopting more targeted treatment strategies. Glucocorticoids play a central role in both conditions. In severe autoimmune diseases, high‐dose short‐term pulse therapy is often used to rapidly control inflammation and reduce cumulative dosage [41]. For pathological scar treatment, injectable corticosteroids are often combined with local injections of 5‐fluorouracil (5‐FU), laser therapy, and surgery to enhance therapeutic effects, offering advantages over monotherapy [42]. In both autoimmune diseases and pathological scar treatment, immunosuppressants have become standard therapeutic strategies. Currently, more targeted immunosuppressants are used clinically to reduce systemic side effects. For example, cyclosporine inhibits the production and proliferation of T cells by blocking interleukin‐2 (IL‐2) in T cells [43]. Rapamycin, by inhibiting the PI3K/AKT/mTOR signaling pathway, blocks the growth and differentiation of immune cells [44]. In cell experiments, the immunosuppressant rapamycin has been shown to inhibit the proliferation of keloid fibroblasts, suggesting it as a new target for inhibiting fibrosis [45]. Despite these similarities, autoimmune diseases and pathological scars exhibit significant differences in treatment goals, drug selection, mechanisms of action, and adverse effects. Pathological scar treatment focuses more on local inhibition of fibroblast proliferation and collagen synthesis, often combined with laser or surgical interventions. Autoimmune diseases, however, emphasize systemic suppression of abnormal immune responses.

This study has several strengths. First, it used a bidirectional two‐sample MR design, which reduces confounding and reverse causation relative to conventional observational studies. Second, we performed multiple sensitivity analyses to evaluate heterogeneity, pleiotropy, and influential SNPs. Third, the study provides a framework for exploring potential shared genetic signals between autoimmune diseases and pathological scars. However, these strengths do not overcome the fact that the main findings did not survive FDR correction.

Several limitations should be considered. First, none of the 40 primary tests survived FDR correction, so the nominal results do not constitute robust causal evidence. Second, the relaxed *p* < 5 × 10^−6^ threshold for scar instruments increased the risk of weak‐instrument bias, horizontal pleiotropy, and winner's curse despite all retained F statistics exceeding 10. Third, the hypertrophic scar–celiac disease estimate used only two SNPs, precluding reliable MR‐Egger, heterogeneity, and outlier diagnostics. Fourth, MR‐Egger and MR‐PRESSO have limited power and cannot exclude all forms of pleiotropy. Fifth, exact sample overlap could not be quantified and strong instruments cannot eliminate overlap bias. Sixth, the relatively small numbers of keloid and hypertrophic‐scar cases probably limited power to detect modest effects; a formal minimum detectable effect could not be estimated consistently because exposure variance explained and comparable trait‐scale parameters were unavailable for every dataset. Null estimates should therefore not be interpreted as proof of absence. Seventh, phenotype definitions and diagnostic accuracy may differ across cohorts, and misclassification between keloids and hypertrophic scars is possible. Eighth, the predominantly European ancestry restricts generalizability, which is particularly important because pathological‐scar prevalence and severity vary substantially by ancestry. Ninth, colocalization was not performed; without it, associations arising from distinct variants in linkage disequilibrium cannot be distinguished from a shared causal variant. Future multivariable MR could also help separate mediation through correlated autoimmune traits or inflammatory pathways. Finally, MR estimates concern inherited genetic liability and do not represent the clinical effects of disease duration, severity, treatment, or environmental exposures.

In conclusion, this bidirectional MR study identified three nominal associations, but none remained significant after correction for 40 primary tests. The results therefore do not provide robust evidence that genetic liability to the studied autoimmune diseases causes pathological scars or vice versa. The study's main contribution is an initial genetic framework for investigating potentially shared inflammatory and fibrotic mechanisms. Larger scar GWAS, stronger instruments, colocalization, multivariable MR, and replication in ancestrally diverse populations are required before causal or clinical conclusions can be drawn.

## Author Contributions

Xianglin Dong proposed the conception, designed the study and supervised the whole analysis. Xueqi Wang acquired the data and performed statistical analysis. Xueqi Wang drafted the article. Xianglin Dong contributed to the data acquisition and checked the statistical analysis.

## Funding

This work was supported by grants from General Program of Natural Science Foundation of Xinjiang Uygur Autonomous Region (2023D01C101).

## Ethics Statement

The authors have nothing to report.

## Consent

The authors have nothing to report.

## Conflicts of Interest

The authors declare no conflicts of interest.

## Supporting information


**Table S1:** Characteristics of SNPs associated with hypertrophic scar and autoimmune diseases (*p* < 5 × 10^−6^).

**Table S2:** Characteristics of SNPs associated with keloid and autoimmune diseases (*p* < 5 × 10^−6^).

**Table S3:** Characteristics of SNPs associated with autoimmune diseases and hypertrophic scar (*p* < 5 × 10^−8^).

**Table S4:** Characteristics of SNPs associated with autoimmune diseases and keloid (*p* < 5 × 10^−8^).

**Table S5:** Mendelian randomization assessment of the association between hypertrophic scar and autoimmune diseases (*p* < 5 × 10^−6^).

**Table S6:** Mendelian randomization assessment of the association between keloid and autoimmune diseases (*p* < 5 × 10^−6^).

**Table S7:** Mendelian randomization assessment of the association between autoimmune diseases and hypertrophic scar (*p* < 5 × 10^−8^).

**Table S8:** Mendelian randomization assessment of the association between autoimmune diseases and keloid (*p* < 5 × 10^−8^).

**Data S1:** STROBE‐MR checklist of recommended items to address in reports of Mendelian randomization studies.

**Figure S1:** (A) Forest plot for the effect of hypertrophic scar on systemic lupus erythematosus. (B) Leave‐one‐out sensitivity analysis plot for the effect of hypertrophic scar on systemic lupus erythematosus. (C) Scatter plot for the effect of hypertrophic scar on systemic lupus erythematosus.
**Figure S2:** (A) Forest plot for the effect of hypertrophic scar on psoriasis. (B) Leave‐one‐out sensitivity analysis plot for the effect of hypertrophic scar on psoriasis. (C) Scatter plot for the effect of hypertrophic scar on psoriasis.
**Figure S3:** (A) Forest plot for the effect of hypertrophic scar on type 1 diabetes. (B) Leave‐one‐out sensitivity analysis plot for the effect of hypertrophic scar on type 1 diabetes. (C) Scatter plot for the effect of hypertrophic scar on type 1 diabetes.
**Figure S4:** (A) Forest plot for the effect of hypertrophic scar on primary biliary cholangitis. (B) Leave‐one‐out sensitivity analysis plot for the effect of hypertrophic scar on primary biliary cholangitis. (C) Scatter plot for the effect of hypertrophic scar on primary biliary cholangitis.
**Figure S5:** (A) Forest plot for the effect of hypertrophic scar on rheumatoid arthritis. (B) Leave‐one‐out sensitivity analysis plot for the effect of hypertrophic scar on rheumatoid arthritis. (C) Scatter plot for the effect of hypertrophic scar on rheumatoid arthritis.
**Figure S6:** (A) Forest plot for the effect of hypertrophic scar on ulcerative colitis. (B) Leave‐one‐out sensitivity analysis plot for the effect of hypertrophic scar on ulcerative colitis. (C) Scatter plot for the effect of hypertrophic scar on ulcerative colitis.
**Figure S7:** (A) Forest plot for the effect of hypertrophic scar on celiac disease. (B) N/A (Not Applicable). (C) Scatter plot for the effect of hypertrophic scar on celiac disease.
**Figure S8:** (A) Forest plot for the effect of hypertrophic scar on Crohn's disease. (B) Leave‐one‐out sensitivity analysis plot for the effect of hypertrophic scar on Crohn's disease. (C) Scatter plot for the effect of hypertrophic scar on Crohn's disease.
**Figure S9:** (A) Forest plot for the effect of hypertrophic scar on multiple sclerosis. (B) Leave‐one‐out sensitivity analysis plot for the effect of hypertrophic scar on multiple sclerosis. (C) Scatter plot for the effect of hypertrophic scar on multiple sclerosis.
**Figure S10:** (A) Forest plot for the effect of hypertrophic scar on ankylosing spondylitis. (B) Leave‐one‐out sensitivity analysis plot for the effect of hypertrophic scar on ankylosing spondylitis. (C) Scatter plot for the effect of hypertrophic scar on ankylosing spondylitis.

**Figure S11:** (A) Forest plot for the effect of keloid SNPs on systemic lupus erythematosus. (B) Leave‐one‐out sensitivity analysis plot for the effect of keloid SNPs on systemic lupus erythematosus. (C) Scatter plot for the effect of keloid SNPs on systemic lupus erythematosus.
**Figure S12:** (A) Forest plot for the effect of keloid SNPs on psoriasis. (B) Leave‐one‐out sensitivity analysis plot for the effect of keloid SNPs on psoriasis. (C) Scatter plot for the effect of keloid SNPs on psoriasis.
**Figure S13:** (A) Forest plot for the effect of keloid SNPs on type 1 diabetes. (B) Leave‐one‐out sensitivity analysis plot for the effect of keloid SNPs on type 1 diabetes. (C) Scatter plot for the effect of keloid SNPs on type 1 diabetes.
**Figure S14:** (A) Forest plot for the effect of keloid SNPs on primary biliary cholangitis. (B) Leave‐one‐out sensitivity analysis plot for the effect of keloid SNPs on primary biliary cholangitis. (C) Scatter plot for the effect of keloid SNPs on primary biliary cholangitis.
**Figure S15:** (A) Forest plot for the effect of keloid SNPs on rheumatoid arthritis. (B) Leave‐one‐out sensitivity analysis plot for the effect of keloid SNPs on rheumatoid arthritis. (C) Scatter plot for the effect of keloid SNPs on rheumatoid arthritis.
**Figure S16:** (A) Forest plot for the effect of keloid SNPs on ulcerative colitis. (B) Leave‐one‐out sensitivity analysis plot for the effect of keloid SNPs on ulcerative colitis. (C) Scatter plot for the effect of keloid SNPs on ulcerative colitis.
**Figure S17:** (A) Forest plot for the effect of keloid SNPs on celiac disease. (B) Leave‐one‐out sensitivity analysis plot for the effect of keloid SNPs on celiac disease. (C) Scatter plot for the effect of keloid SNPs on celiac disease.
**Figure S18:** (A) Forest plot for the effect of keloid SNPs on Crohn's disease. (B) Leave‐one‐out sensitivity analysis plot for the effect of keloid SNPs on Crohn's disease. (C) Scatter plot for the effect of keloid SNPs on Crohn's disease.
**Figure S19:** (A) Forest plot for the effect of keloid SNPs on multiple sclerosis. (B) Leave‐one‐out sensitivity analysis plot for the effect of keloid SNPs on multiple sclerosis. (C) Scatter plot for the effect of keloid SNPs on multiple sclerosis.
**Figure S20:** (A) Forest plot for the effect of keloid SNPs on ankylosing spondylitis. (B) Leave‐one‐out sensitivity analysis plot for the effect of keloid SNPs on ankylosing spondylitis. (C) Scatter plot for the effect of keloid SNPs on ankylosing spondylitis.

**Figure S21:** (A) Forest plot for the effect of systemic lupus erythematosus SNPs on hypertrophic scar. (B) Leave‐one‐out sensitivity analysis plot for the effect of systemic lupus erythematosus SNPs on hypertrophic scar. (C) Scatter plot for the effect of systemic lupus erythematosus SNPs on hypertrophic scar.
**Figure S22:** (A) Forest plot for the effect of psoriasis SNPs on hypertrophic scar. (B) Leave‐one‐out sensitivity analysis plot for the effect of psoriasis SNPs on hypertrophic scar. (C) Scatter plot for the effect of psoriasis SNPs on hypertrophic scar.
**Figure S23:** (A) Forest plot for the effect of type 1 diabetes SNPs on hypertrophic scar. (B) Leave‐one‐out sensitivity analysis plot for the effect of type 1 diabetes SNPs on hypertrophic scar. (C) Scatter plot for the effect of type 1 diabetes SNPs on hypertrophic scar.
**Figure S24:** (A) Forest plot for the effect of primary biliary cholangitis SNPs on hypertrophic scar. (B) Leave‐one‐out sensitivity analysis plot for the effect of primary biliary cholangitis SNPs on hypertrophic scar. (C) Scatter plot for the effect of primary biliary cholangitis SNPs on hypertrophic scar.
**Figure S25:** (A) Forest plot for the effect of rheumatoid arthritis SNPs on hypertrophic scar. (B) Leave‐one‐out sensitivity analysis plot for the effect of rheumatoid arthritis SNPs on hypertrophic scar. (C) Scatter plot for the effect of rheumatoid arthritis SNPs on hypertrophic scar.
**Figure S26:** (A) Forest plot for the effect of ulcerative colitis SNPs on hypertrophic scar. (B) Leave‐one‐out sensitivity analysis plot for the effect of ulcerative colitis SNPs on hypertrophic scar. (C) Scatter plot for the effect of ulcerative colitis SNPs on hypertrophic scar.
**Figure S27:** (A) Forest plot for the effect of celiac disease SNPs on hypertrophic scar. (B) Leave‐one‐out sensitivity analysis plot for the effect of celiac disease SNPs on hypertrophic scar. (C) Scatter plot for the effect of celiac disease SNPs on hypertrophic scar.
**Figure S28:** (A) Forest plot for the effect of Crohn's disease SNPs on hypertrophic scar. (B) Leave‐one‐out sensitivity analysis plot for the effect of Crohn's disease SNPs on hypertrophic scar. (C) Scatter plot for the effect of Crohn's disease SNPs on hypertrophic scar.
**Figure S29:** (A) Forest plot for the effect of multiple sclerosis SNPs on hypertrophic scar. (B) Leave‐one‐out sensitivity analysis plot for the effect of multiple sclerosis SNPs on hypertrophic scar. (C) Scatter plot for the effect of multiple sclerosis SNPs on hypertrophic scar.
**Figure S30:** (A) Forest plot for the effect of ankylosing spondylitis SNPs on hypertrophic scar. (B) NA. (C) Scatter plot for the effect of ankylosing spondylitis SNPs on hypertrophic scar.

**Figure S31:** (A) Forest plot for the effect of systemic lupus erythematosus SNPs on keloid. (B) Leave‐one‐out sensitivity analysis plot for the effect of systemic lupus erythematosus SNPs on keloid. (C) Scatter plot for the effect of systemic lupus erythematosus SNPs on keloid.
**Figure S32:** (A) Forest plot for the effect of psoriasis SNPs on keloid. (B) Leave‐one‐out sensitivity analysis plot for the effect of psoriasis SNPs on keloid. (C) Scatter plot for the effect of psoriasis SNPs on keloid.
**Figure S33:** (A) Forest plot for the effect of type 1 diabetes SNPs on keloid. (B) Leave‐one‐out sensitivity analysis plot for the effect of type 1 diabetes SNPs on keloid. (C) Scatter plot for the effect of type 1 diabetes SNPs on keloid.
**Figure S34:** (A) Forest plot for the effect of primary biliary cholangitis SNPs on keloid. (B) Leave‐one‐out sensitivity analysis plot for the effect of primary biliary cholangitis SNPs on keloid. (C) Scatter plot for the effect of primary biliary cholangitis SNPs on keloid.
**Figure S35:** (A) Forest plot for the effect of rheumatoid arthritis SNPs on keloid. (B) Leave‐one‐out sensitivity analysis plot for the effect of rheumatoid arthritis SNPs on keloid. (C) Scatter plot for the effect of rheumatoid arthritis SNPs on keloid.
**Figure S36:** (A) Forest plot for the effect of ulcerative colitis SNPs on keloid. (B) Leave‐one‐out sensitivity analysis plot for the effect of ulcerative colitis SNPs on keloid. (C) Scatter plot for the effect of ulcerative colitis SNPs on keloid.
**Figure S37:** (A) Forest plot for the effect of celiac disease SNPs on keloid. (B) Leave‐one‐out sensitivity analysis plot for the effect of celiac disease SNPs on keloid. (C) Scatter plot for the effect of celiac disease SNPs on keloid.
**Figure S38:** (A) Forest plot for the effect of Crohn's disease SNPs on keloid. (B) Leave‐one‐out sensitivity analysis plot for the effect of Crohn's disease SNPs on keloid. (C) Scatter plot for the effect of Crohn's disease SNPs on keloid.
**Figure S39:** (A) Forest plot for the effect of multiple sclerosis SNPs on keloid. (B) Leave‐one‐out sensitivity analysis plot for the effect of multiple sclerosis SNPs on keloid. (C) Scatter plot for the effect of multiple sclerosis SNPs on keloid.
**Figure S40:** (A) Forest plot for the effect of ankylosing spondylitis SNPs on keloid. (B) Leave‐one‐out sensitivity analysis plot for the effect of ankylosing spondylitis SNPs on keloid. (C) Scatter plot for the effect of ankylosing spondylitis SNPs on keloid.

## Data Availability

The raw data and original contributions supporting the conclusions of this article are available in the Supporting Information—S1 provided. For any additional datasets or specific inquiries, please contact the corresponding author. The summary statistics utilized in our analysis are publicly accessible from the FinnGen study (https://www.finngen.fi/en), the IEU OpenGWAS project (https://gwas.mrcieu.ac.uk/), and the GWAS Catalog (https://www.ebi.ac.uk/gwas/).
