# Supplementary material for: Exploring Genetic Associations Between Autoimmune Diseases and Pathological Scars: A Bidirectional Mendelian Randomization Analysis
Source: J Cosmet Dermatol. 2026 Aug 19;25(8):e71128. doi: 10.1111/jocd.71128 (PMC13490626; doi:10.1111/jocd.71128)
Supplement: Supplementary file 1 [file JOCD-25-e71128-s001.docx]

**Supplementary Fig31:** (A) Forest plot for the effect of systemic lupus erythematosus SNPs on keloid. (B) Leave-one-out sensitivity analysis plot for the effect of systemic lupus erythematosus SNPs on keloid. (C) Scatter plot for the effect of systemic lupus erythematosus SNPs on keloid.

**Supplementary Fig32:** (A) Forest plot for the effect of psoriasis SNPs on keloid. (B) Leave-one-out sensitivity analysis plot for the effect of psoriasis SNPs on keloid. (C) Scatter plot for the effect of psoriasis SNPs on keloid.

**Supplementary Fig33:** (A) Forest plot for the effect of type 1 diabetes SNPs on keloid. (B) Leave-one-out sensitivity analysis plot for the effect of type 1 diabetes SNPs on keloid. (C) Scatter plot for the effect of type 1 diabetes SNPs on keloid.

**Supplementary Fig34:** (A) Forest plot for the effect of primary biliary cholangitis SNPs on keloid. (B) Leave-one-out sensitivity analysis plot for the effect of primary biliary cholangitis SNPs on keloid. (C) Scatter plot for the effect of primary biliary cholangitis SNPs on keloid.

**Supplementary Fig35:** (A) Forest plot for the effect of rheumatoid arthritis SNPs on keloid. (B) Leave-one-out sensitivity analysis plot for the effect of rheumatoid arthritis SNPs on keloid. (C) Scatter plot for the effect of rheumatoid arthritis SNPs on keloid.

**Supplementary Fig36:** (A) Forest plot for the effect of ulcerative colitis SNPs on keloid. (B) Leave-one-out sensitivity analysis plot for the effect of ulcerative colitis SNPs on keloid. (C) Scatter plot for the effect of ulcerative colitis SNPs on keloid.

**Supplementary Fig37:** (A) Forest plot for the effect of celiac disease SNPs on keloid. (B) Leave-one-out sensitivity analysis plot for the effect of celiac disease SNPs on keloid. (C) Scatter plot for the effect of celiac disease SNPs on keloid.

**Supplementary Fig38:** (A) Forest plot for the effect of Crohn's disease SNPs on keloid. (B) Leave-one-out sensitivity analysis plot for the effect of Crohn's disease SNPs on keloid. (C) Scatter plot for the effect of Crohn's disease SNPs on keloid.

**Supplementary Fig 39:** (A) Forest plot for the effect of multiple sclerosis SNPs on keloid. (B) Leave-one-out sensitivity analysis plot for the effect of multiple sclerosis SNPs on keloid. (C) Scatter plot for the effect of multiple sclerosis SNPs on keloid.

**Supplementary Fig40:** (A) Forest plot for the effect of ankylosing spondylitis SNPs on keloid. (B) Leave-one-out sensitivity analysis plot for the effect of ankylosing spondylitis SNPs on keloid. (C) Scatter plot for the effect of ankylosing spondylitis SNPs on keloid.
