# Supplementary material for: Exploring Genetic Associations Between Autoimmune Diseases and Pathological Scars: A Bidirectional Mendelian Randomization Analysis
Source: J Cosmet Dermatol. 2026 Aug 19;25(8):e71128. doi: 10.1111/jocd.71128 (PMC13490626; doi:10.1111/jocd.71128)
Supplement: Supplementary file 2 [file JOCD-25-e71128-s004.docx]

**Supplementary Fig 21:** (A) Forest plot for the effect of systemic lupus erythematosus SNPs on hypertrophic scar. (B) Leave-one-out sensitivity analysis plot for the effect of systemic lupus erythematosus SNPs on hypertrophic scar. (C) Scatter plot for the effect of systemic lupus erythematosus SNPs on hypertrophic scar.

**Supplementary Fig 22:** (A) Forest plot for the effect of psoriasis SNPs on hypertrophic scar. (B) Leave-one-out sensitivity analysis plot for the effect of psoriasis SNPs on hypertrophic scar. (C) Scatter plot for the effect of psoriasis SNPs on hypertrophic scar.

**Supplementary Fig 23:** (A) Forest plot for the effect of type 1 diabetes SNPs on hypertrophic scar. (B) Leave-one-out sensitivity analysis plot for the effect of type 1 diabetes SNPs on hypertrophic scar. (C) Scatter plot for the effect of type 1 diabetes SNPs on hypertrophic scar.

**Supplementary Fig 24:** (A) Forest plot for the effect of primary biliary cholangitis SNPs on hypertrophic scar. (B) Leave-one-out sensitivity analysis plot for the effect of primary biliary cholangitis SNPs on hypertrophic scar. (C) Scatter plot for the effect of primary biliary cholangitis SNPs on hypertrophic scar.

**Supplementary Fig 25:** (A) Forest plot for the effect of rheumatoid arthritis SNPs on hypertrophic scar. (B) Leave-one-out sensitivity analysis plot for the effect of rheumatoid arthritis SNPs on hypertrophic scar. (C) Scatter plot for the effect of rheumatoid arthritis SNPs on hypertrophic scar.

**Supplementary Fig 26:** (A) Forest plot for the effect of ulcerative colitis SNPs on hypertrophic scar. (B) Leave-one-out sensitivity analysis plot for the effect of ulcerative colitis SNPs on hypertrophic scar. (C) Scatter plot for the effect of ulcerative colitis SNPs on hypertrophic scar.

**Supplementary Fig 27:** (A) Forest plot for the effect of celiac disease SNPs on hypertrophic scar. (B) Leave-one-out sensitivity analysis plot for the effect of celiac disease SNPs on hypertrophic scar. (C) Scatter plot for the effect of celiac disease SNPs on hypertrophic scar.

**Supplementary Fig 28:** (A) Forest plot for the effect of Crohn's disease SNPs on hypertrophic scar. (B) Leave-one-out sensitivity analysis plot for the effect of Crohn's disease SNPs on hypertrophic scar. (C) Scatter plot for the effect of Crohn's disease SNPs on hypertrophic scar.

**Supplementary Fig 29:** (A) Forest plot for the effect of multiple sclerosis SNPs on hypertrophic scar. (B) Leave-one-out sensitivity analysis plot for the effect of multiple sclerosis SNPs on hypertrophic scar. (C) Scatter plot for the effect of multiple sclerosis SNPs on hypertrophic scar.

**Supplementary Fig 30:** (A) Forest plot for the effect of ankylosing spondylitis SNPs on hypertrophic scar. (B) NA. (C) Scatter plot for the effect of ankylosing spondylitis SNPs on hypertrophic scar.
