# Supplementary material for: Exploring Genetic Associations Between Autoimmune Diseases and Pathological Scars: A Bidirectional Mendelian Randomization Analysis
Source: J Cosmet Dermatol. 2026 Aug 19;25(8):e71128. doi: 10.1111/jocd.71128 (PMC13490626; doi:10.1111/jocd.71128)
Supplement: Supplementary file 3 [file JOCD-25-e71128-s006.docx]

**Supplementary Fig1:**(A) Forest plot for the effect of hypertrophic scar on systemic lupus erythematosus. (B) Leave-one-out sensitivity analysis plot for the effect of hypertrophic scar on systemic lupus erythematosus. (C) Scatter plot for the effect of hypertrophic scar on systemic lupus erythematosus.

**Supplementary Fig2:**(A) Forest plot for the effect of hypertrophic scar on psoriasis. (B) Leave-one-out sensitivity analysis plot for the effect of hypertrophic scar on psoriasis. (C) Scatter plot for the effect of hypertrophic scar on psoriasis.

**Supplementary Fig3:**(A) Forest plot for the effect of hypertrophic scar on type 1 diabetes. (B) Leave-one-out sensitivity analysis plot for the effect of hypertrophic scar on type 1 diabetes. (C) Scatter plot for the effect of hypertrophic scar on type 1 diabetes.

**Supplementary Fig4:**(A) Forest plot for the effect of hypertrophic scar on primary biliary cholangitis. (B) Leave-one-out sensitivity analysis plot for the effect of hypertrophic scar on primary biliary cholangitis. (C) Scatter plot for the effect of hypertrophic scar on primary biliary cholangitis.

**Supplementary Fig5:**(A) Forest plot for the effect of hypertrophic scar on rheumatoid arthritis. (B) Leave-one-out sensitivity analysis plot for the effect of hypertrophic scar on rheumatoid arthritis. (C) Scatter plot for the effect of hypertrophic scar on rheumatoid arthritis.

**Supplementary Fig6:**(A) Forest plot for the effect of hypertrophic scar on ulcerative colitis. (B) Leave-one-out sensitivity analysis plot for the effect of hypertrophic scar on ulcerative colitis. (C) Scatter plot for the effect of hypertrophic scar on ulcerative colitis.

**Supplementary Fig7:**(A) Forest plot for the effect of hypertrophic scar on celiac disease. (B) N/A (Not Applicable). (C) Scatter plot for the effect of hypertrophic scar on celiac disease.

**Supplementary Fig8:**(A) Forest plot for the effect of hypertrophic scar on Crohn's disease. (B) Leave-one-out sensitivity analysis plot for the effect of hypertrophic scar on Crohn's disease. (C) Scatter plot for the effect of hypertrophic scar on Crohn's disease.

**Supplementary Fig9:**(A) Forest plot for the effect of hypertrophic scar on multiple sclerosis. (B) Leave-one-out sensitivity analysis plot for the effect of hypertrophic scar on multiple sclerosis. (C) Scatter plot for the effect of hypertrophic scar on multiple sclerosis.

**Supplementary Fig10:**(A) Forest plot for the effect of hypertrophic scar on ankylosing spondylitis. (B) Leave-one-out sensitivity analysis plot for the effect of hypertrophic scar on ankylosing spondylitis. (C) Scatter plot for the effect of hypertrophic scar on ankylosing spondylitis.
