# Supplementary material for: Exploring Genetic Associations Between Autoimmune Diseases and Pathological Scars: A Bidirectional Mendelian Randomization Analysis
Source: J Cosmet Dermatol. 2026 Aug 19;25(8):e71128. doi: 10.1111/jocd.71128 (PMC13490626; doi:10.1111/jocd.71128)
Supplement: Supplementary file 4 [file JOCD-25-e71128-s007.docx]

**Supplementary Fig 11:** (A) Forest plot for the effect of keloid SNPs on systemic lupus erythematosus. (B) Leave-one-out sensitivity analysis plot for the effect of keloid SNPs on systemic lupus erythematosus. (C) Scatter plot for the effect of keloid SNPs on systemic lupus erythematosus.

**Supplementary Fig 12:** (A) Forest plot for the effect of keloid SNPs on psoriasis. (B) Leave-one-out sensitivity analysis plot for the effect of keloid SNPs on psoriasis. (C) Scatter plot for the effect of keloid SNPs on psoriasis.

**Supplementary Fig 13:** (A) Forest plot for the effect of keloid SNPs on type 1 diabetes. (B) Leave-one-out sensitivity analysis plot for the effect of keloid SNPs on type 1 diabetes. (C) Scatter plot for the effect of keloid SNPs on type 1 diabetes.

**Supplementary Fig 14:** (A) Forest plot for the effect of keloid SNPs on primary biliary cholangitis. (B) Leave-one-out sensitivity analysis plot for the effect of keloid SNPs on primary biliary cholangitis. (C) Scatter plot for the effect of keloid SNPs on primary biliary cholangitis.

**Supplementary Fig15:** (A) Forest plot for the effect of keloid SNPs on rheumatoid arthritis. (B) Leave-one-out sensitivity analysis plot for the effect of keloid SNPs on rheumatoid arthritis. (C) Scatter plot for the effect of keloid SNPs on rheumatoid arthritis.

**Supplementary Fig 16:** (A) Forest plot for the effect of keloid SNPs on ulcerative colitis. (B) Leave-one-out sensitivity analysis plot for the effect of keloid SNPs on ulcerative colitis. (C) Scatter plot for the effect of keloid SNPs on ulcerative colitis.

**Supplementary Fig 17:** (A) Forest plot for the effect of keloid SNPs on celiac disease. (B) Leave-one-out sensitivity analysis plot for the effect of keloid SNPs on celiac disease. (C) Scatter plot for the effect of keloid SNPs on celiac disease.

**Supplementary Fig 18:** (A) Forest plot for the effect of keloid SNPs on Crohn's disease. (B) Leave-one-out sensitivity analysis plot for the effect of keloid SNPs on Crohn's disease. (C) Scatter plot for the effect of keloid SNPs on Crohn's disease.

**Supplementary Fig 19:** (A) Forest plot for the effect of keloid SNPs on multiple sclerosis. (B) Leave-one-out sensitivity analysis plot for the effect of keloid SNPs on multiple sclerosis. (C) Scatter plot for the effect of keloid SNPs on multiple sclerosis.

**Supplementary Fig 20:** (A) Forest plot for the effect of keloid SNPs on ankylosing spondylitis. (B) Leave-one-out sensitivity analysis plot for the effect of keloid SNPs on ankylosing spondylitis. (C) Scatter plot for the effect of keloid SNPs on ankylosing spondylitis.
